# Knockdown of lincRNA PADNA promotes bupivacaine-induced neurotoxicity by miR-194/FBXW7 axis

**DOI:** 10.1186/s10020-020-00209-8

**Published:** 2020-08-13

**Authors:** Fan Yuning, Chen Liang, Wang Tenghuan, Nan Zhenhua, Gong Shengkai

**Affiliations:** 1 Department of Anesthesiology, Pain and Perioperative Medicine, The First Affiliated Hospital of Zhengzhou University, Henan, China; 2 Department of Anesthesiology, The Fourth Hospital of Shijiazhuang, Shijiazhuang, Hebei China

**Keywords:** lincRNA, PADNA, miR-194, FBXW7

## Abstract

**Background:**

The aim of the study was to explore the function and mechanism of lincRNA PADNA in bupivacaine-induced neurotoxicity.

**Methods:**

Mouse DRG neurons were cultured in vitro and treated with bupivacaine to establish a neurotoxicity model. Caspase3 activity, cell viability, and TUNEL assays were analyzed to assess the role of lincRNA PADNA. A dual-luciferase reporter assay was used to determine the binding target of lincRNA PANDA.

**Results:**

The expression of lincRNA PADNA was significantly increased with increasing concentrations of bupivacaine. Functional analysis revealed that knockdown of lincRNA PADNA increased caspase3 activity and inhibited cell viability. Western blot analysis showed that knockdown of lincRNA PADNA promoted cleaved caspase3 levels. We also revealed that lincRNA PADNA may bind with miR-194. Knockdown of miR-194 rescued the function of lincRNA PADNA, suggesting that lincRNA PADNA may sponge miR-194. In addition, we provided new evidence that the lincRNA PADNA/miR-194/FBXW7 axis plays an important role in the neurotoxicity process.

**Conclusion:**

We performed comprehensive experiments to verify the function and mechanism of lincRNA PADNA in bupivacaine-induced neurotoxicity. Our study provides new evidence and clues for the prevention of neurotoxicity.

## Introduction

Bupivacaine is one of the most commonly used anesthetics for local infiltration anesthesia (Radwan et al. 2002; Guo et al. 2017; Chalkiadis et al. 2016). Studies have demonstrated that the adverse drug reactions (ADRs) to bupivacaine are mainly limited to the central nervous system (CNS) and cardiovascular system because of the systemic absorption of bupivacaine (Ammar and Mahmoud 2012; Kurihara et al. 2008). During the past few decades, bupivacaine has been found to be neurotoxic in the setting of local injection, causing symptoms such as paralysis, paresthesia, hypoventilation, and fecal and urinary incontinence (Xianjie et al. 2013; Traore et al. 2006; Helal et al. 2016; Ferrillo 2016). The side effects of bupivacaine have aroused enormous interest and attention, and a great number of studies have been conducted to elucidate the mechanism of bupivacaine-induced neurotoxicity and to find ways to prevent or target these side effects (Xianjie et al. 2013). However, the results have been difficult to interpret, and the mechanism of bupivacaine-induced neurotoxicity remains unclear.

Efforts have been made to investigate the association between bupivacaine-induced neurotoxicity and noncoding RNAs (ncRNAs) (Wang et al. 2015). NcRNAs are RNA molecules that cannot be translated into protein (Liu et al. 2016). There are different types of ncRNAs, including transfer RNAs, ribosomal RNAs, microRNAs, and lncRNAs (Yamada et al. 2018; Li et al. 2018). Jiang R found that miR-489-3p could promote bupivacaine-induced apoptosis by regulating the PI3K/AKT pathway (Jiang et al. 2017). LncRNAs are defined as transcripts with lengths exceeding 200 nucleotides (Song et al. 2017), which have been widely studied and found to be abundantly and functionally important in regulating the cell cycle (Liu et al. 2015), cell metabolism (Zhang et al. 2016a) and related diseases such as malignant tumors (Gu et al. 2018). However, the role of lncRNAs in bupivacaine-induced neurotoxicity has rarely been researched.

In the current research, we investigated the long noncoding RNA Gm14012 (named lincRNA PADNA, protect cell death RNA, for its role in protect against cell death). To investigate the underlying mechanism of how lincRNA PADNA participates in bupivacaine-induced neurotoxicity, we conducted bioinformatics analysis, and the results revealed that lincRNA PADNA played a protective role through inhibition of the progression of bupivacaine-induced neurotoxicity by sponging miR-194, which has been reported to inhibit tumor progression (Wu et al. 2014). miR-194 was predicted to target the 3’UTR of the cancer-related protein F-box and WD repeat domain containing 7 (FBXW7) by analysis in StarBase2.0. The current research may provide new targets for inhibiting or reversing bupivacaine-induced neurotoxicity.

## Materials and methods

### Cell culture and treatment

HEK293 cells were stored in our laboratory, and primary dorsal root ganglion (DRG) neurons were isolated from 5-week-old C57BL/6 mice as previously described (Zhang et al. 2016b). Briefly, 5-week-old C57BL/6 mice were anesthetized and sacrificed by cervical dislocation. The L4-L5 portion of the spinal cord was extracted. The dorsal root ganglia were collected and dissociated with 0.25% trypsin (Invitrogen, USA). The cells were washed with 2.5% bovine serum albumin (BSA, Invitrogen, USA) and resuspended in serum-free neurobasal medium (Invitrogen, USA) supplemented with penicillin/streptomycin (40,000 unit/L, Invitrogen, USA) and B-27 serum-free supplement (Invitrogen, USA). To induce neurotoxicity, DRG neurons were treated with various concentrations of bupivacaine (0.5, 1.0, 1.5 or 2.0 mM) for 6 h, 12 h, 24 h, and 48 h.

### Transfection

The knockdown vectors of lincRNA PADNA were constructed by Gene Pharma (Shanghai, China). Empty vectors and vectors with wild-type (WT) or mutant (mut) binding sites for miR-194 were constructed by Gene Pharma (Shanghai, China). The 3′-untranslated region (UTR) of FBXW7, containing wild-type (WT) or mutant (mut) binding sites for miR-194, was amplified and cloned into the pGL3 vector (Promega, Madison, WI) to generate the vector pGL3-WT-FBXW7–3′-UTR or pGL3-mut-FBXW7–3′-UTR. The miR-194 mimic, miR-194 inhibitor, mimic NC, and inhibitor NC were purchased from Shanghai Gene Pharma (Shanghai, China). Briefly, DRG neurons were cotransfected with vectors, miR-194 mimic, miR-194 inhibitor, mimic NC and inhibitor NC by the Lipo3000 reagent (Invitrogen) according to the manufacturer’s protocol. Cells were incubated for 48 h before further research.

### Cell viability assay

DRG neurons were seeded into 96-well plates and treated with bupivacaine (0.5, 1.0, 1.5 or 2.0 mM) for 24 h to establish a bupivacaine-induced neurotoxicity model. Ten microliters of 3-(4,5-dimethylthiazol-2-yl)-2,5-diphenyltetrazolium bromide (MTT) solution (5 mg/mL, Beyotime, Shanghai, China) was added to each well and incubated for 4 h at 37 °C. Cell viability was measured by a microplate reader at 570 nm (Bio-Tek, Winooski, VT).

### Caspase-3 activity

A caspase-3 activity assay kit (Beyotime, Shanghai, China) was used to assess caspase-3 activity according to the manufacturer’s protocol. DRG neurons were lysed and centrifuged. A final concentration of 0.2 mM ADEVD-pNA (caspase-3 substrate) was added to the cell supernatant and incubated for 1 h. Caspase-3 activity was measured by a microplate reader at 405 nm (Bio-Tek, Winooski, VT).

### TUNEL assay

A terminal deoxynucleotidyl transferase-mediated dUTP nick end-labeling (TUNEL) assay was conducted to evaluate the apoptosis of DRG neurons. DRG neurons were incubated with TdT and fluorescein-labeled dUTP for 45 min at 37 °C. Then, a FACSCalibur flow cytometer was used to measure the percentage of apoptotic cells.

### Dual-luciferase reporter assay

Empty vector or vector with the wild-type (WT) or mutant (mut) binding sites for miR-194 was cotransfected with the miR-194 mimic or mimic NC. Luciferase activity was analyzed using a dual-luciferase reporter system following the manufacturer’s protocol. Firefly luciferase activity and Renilla luciferase activity were measured with Multiskan Spectrum (Thermo Fisher, USA). Similarly, empty vector or vector containing the 3′-UTR of FBXW7 with the wild-type or mutant binding sites for miR-194 was cotransfected with the miR-194 mimic or mimic NC. Luciferase activity was analyzed using a dual-luciferase reporter system. Firefly luciferase activity and Renilla luciferase activity were measured with Multiskan Spectrum (Thermo Fisher, USA).

### Real-time PCR

Total RNA was extracted by TRIzol reagent (Thermo Fisher Scientific). RNA reverse transcription was performed using a PrimeScript™ RT reagent Kit with gDNA eraser (RR047A; Takara, Tokyo, Japan), and cDNA was assayed using SYBR® Premix Ex Taq™ (RR420A; Takara, Tokyo, Japan). The data were normalized to β-actin levels and further analyzed by the 2^−ΔΔCT^ method.

### Western blotting

DRG neurons were harvested and lysed by RIPA lysis buffer containing proteinase inhibitor (Roche, USA). Total protein was quantified using a BCA protein assay kit (Pierce, Rockford, IL, USA). Protein samples were resolved by 10% SDS-PAGE gels and transferred to polyvinylidene difluoride membranes. After blocking, the membranes were incubated with primary antibodies against caspase3 (1:1000, Abcam, MA, USA), FBXW7 (1:1000, Abcam, MA, USA) and actin (1:1000, Abcam, MA, USA) at 4 °C overnight, followed by incubation with a peroxidase-conjugated goat anti-rabbit (or mouse) IgG antibody. Immunopositive bands were analyzed using a FluorChem M system (ProteinSimple, San Jose, CA, USA).

### Data analysis

We used SPSS 23.0 to calculate the data values (means ± standard error of the mean). Statistical differences were analyzed by unpaired 2-sided Student’s t test or 1-way ANOVA with Bonferroni correction for multiple comparisons. The statistical significance was set at *P* < 0.05.

## Results

### The establishment of a bupivacaine-induced neurotoxicity model

**Fig. 1:**
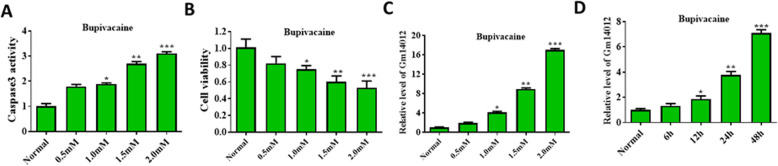
Expression of lincRNA Gm14012 in bupivacaine-induced neurotoxicity. **a** Caspase3 activity of DRG neurons was significantly upregulated with the elevation of concentration of bupivacaine. Mean values: 1.5 mM **b** Cell viability of DRG neurons was significantly decreased with the elevation of concentration of bupivacaine. **c** The expression of lincRNA Gm14012 was significantly upregulated with the elevation of concentration of bupivacaine. **d** The expression of lincRNA Gm14012 was significantly upregulated with the extension of bupivacaine treatment. Data are Mean ± SD with *n* = 3 independent biological cultures. *p < 0.05 and ** *p* < 0.01

To analyze the bupivacaine-induced neurotoxicity process, we first verified the model. Apoptosis and cell viability were analyzed via caspase3 activity and MTT assays. Caspase3 activity increased with increasing concentrations of bupivacaine and reached its highest level at 2.0 mM, the relative increase was about 1.01 ± 0.1, 1.79 ± 0.08, 1.89 ± 0.05, 2.72 ± 0.08, 3.11 ± 0.07 (Fig. 1a). Cell viability was significantly reduced compared with that in the control group. In addition, 2.0 mM bupivacaine reduced cell viability by almost 50%, the relative decrease was about 1.00 ± 0.1, 0.82 ± 0.08, 0.75 ± 0.09, 0.63 ± 0.07, 0.53 ± 0.08 (Fig. 1b). Thus, our results confirmed that bupivacaine induced cell apoptosis.

### Expression of lincRNA Gm14012 in bupivacaine-induced neurotoxicity

We first detected the expression of lincRNA Gm14012 to investigate the possible change. As shown in Fig. 1c, the expression of lincRNA Gm14012 increased with increasing bupivacaine concentration and reached a maximum of an almost 16-fold increase at 2.0 mM, the relative increase was about 1.03 ± 0.1, 1.92 ± 0.18, 4.13 ± 0.24, 8.92 ± 0.28, 17.05 ± 0.27. Similarly, we also detected the expression of lincRNA Gm14012 after exposure to 1.0 mM for different durations. Our results showed that the expression of lincRNA Gm14012 increased with time, the relative increase was 1.01 ± 0.12, 1.33 ± 0.19, 1.87 ± 0.2, 3.77 ± 0.26, 7.09 ± 0.3 (Fig. 1d).

### Function of lincRNA PADNA in bupivacaine-induced neurotoxicity

**Fig. 2:**
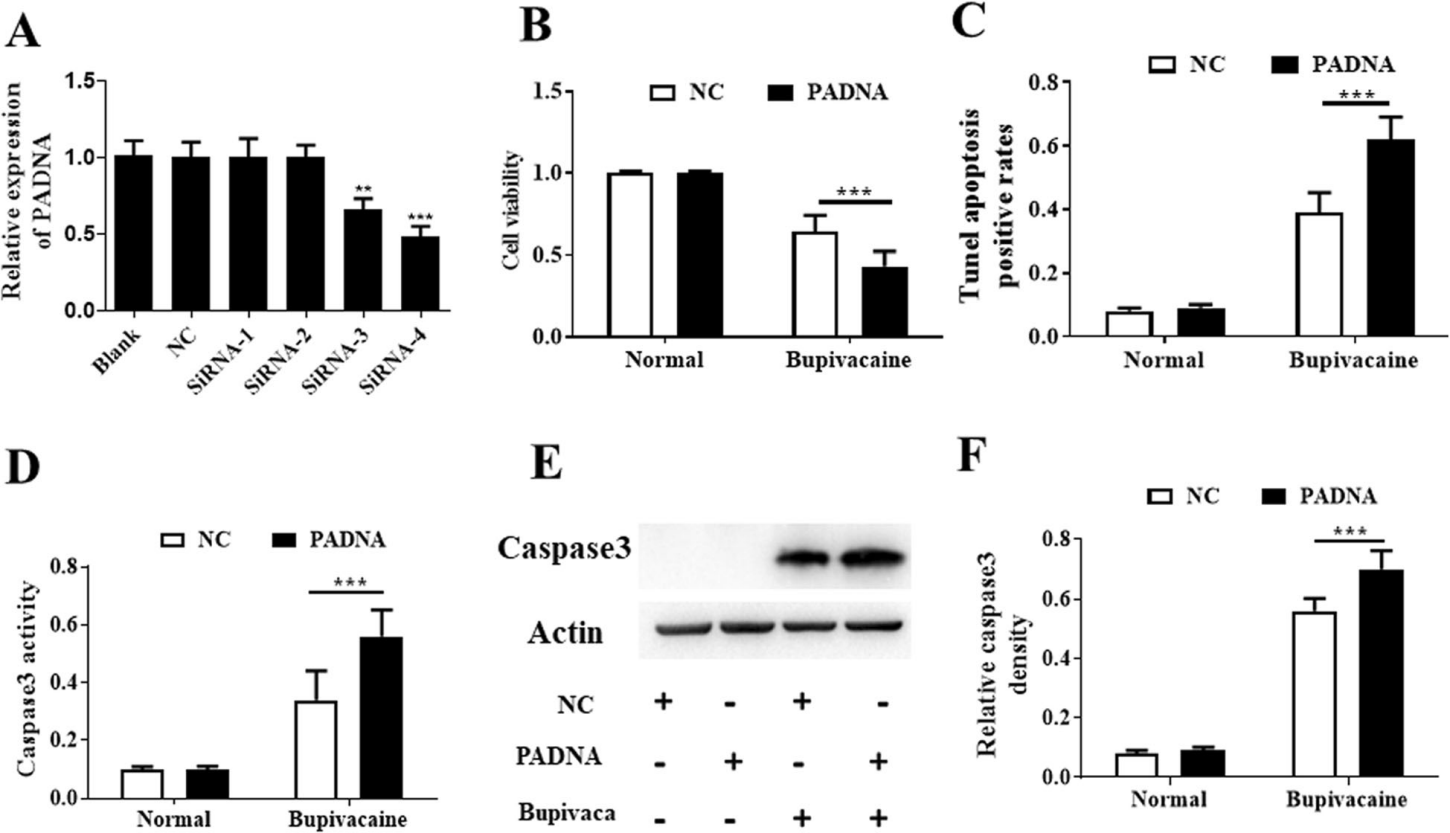
Function of lincRNA PADNA in bupivacaine-induced neurotoxicity. **a** The expressions of lincRNA PADNA were significantly decreased in DRG neurons treated with siRNA3 or siRNA4 when compared with blank group, NC group, DRG neurons treated with siRNA1 or siRNA2. **b** Cell viability was significantly decreased in DRG neurons treated with knockdown vector of lincRNA PADNA. Cells were treated with 1 mM bupivacaine. **c** Percentage of apoptotic of cells was significantly increased in DRG neurons treated with knockdown vector of lincRNA PADNA. Cells were treated with 1 mM bupivacaine. **d** Caspase3 activity was significantly increased in DRG neurons treated with knockdown vector of lincRNA PADNA. Cells were treated with 1 mM bupivacaine. **e**, **f** The expression of Caspase3 protein was significantly increased in DRG neurons treated with knockdown vector of lincRNA PADNA. Cells were treated with 1 mM bupivacaine. Data are Mean ± SD with *n* = 3 independent biological cultures. *p < 0.05 and ** *p* < 0.01

We constructed a knockdown vector to assess the function of lincRNA Gm14012. As shown in Fig. 2a, the expression of lincRNA Gm14012 was significantly downregulated, the relative decrease was about 0.66, 0.48. Next, we used the MTT assay to assess the function of lincRNA Gm14012. Interestingly, knockdown of lincRNA Gm14012 significantly promoted cell death and reduced cell viability after exposure to bupivacaine, the relative decrease was about 0.64 ± 0.1, 0.43 ± 0.08 (Fig. 2b). To date, there has been no report on lincRNA Gm14012 in bupivacaine-induced neurotoxicity. Thus, we named this lincRNA PADNA (protect cell death RNA). To further explore the function of lincRNA PADNA in bupivacaine-induced neurotoxicity, we performed comprehensive analyses, such as TUNEL assay, caspase3 activity assay, and western blotting. TUNEL assays were used to assess cell apoptosis. There was no significant difference between the two groups under normal conditions; however, when cells were exposed to 1.0 mM bupivacaine, knockdown of lincRNA PADNA significantly increased the cell apoptosis rates, suggesting that knockdown of lincRNA PADNA promoted cell death, the relative increase was about 0.41 ± 0.07, 0.63 ± 0.06 (Fig. 2c). In addition, we measured caspase3 activity using a kit. Our results suggested that downregulation of lincRNA PADNA significantly increased caspase3 activity, the relative increase was about 0.34 ± 0.09, 0.56 ± 0.08 (Fig. 2d). Similar results were obtained from western blotting (Fig. 2e). Overall, our experiments demonstrated that lincRNA PADNA played a protective role in bupivacaine-induced neurotoxicity.

### Preliminary analysis of the mechanism of lincRNA PADNA

**Fig. 3:**
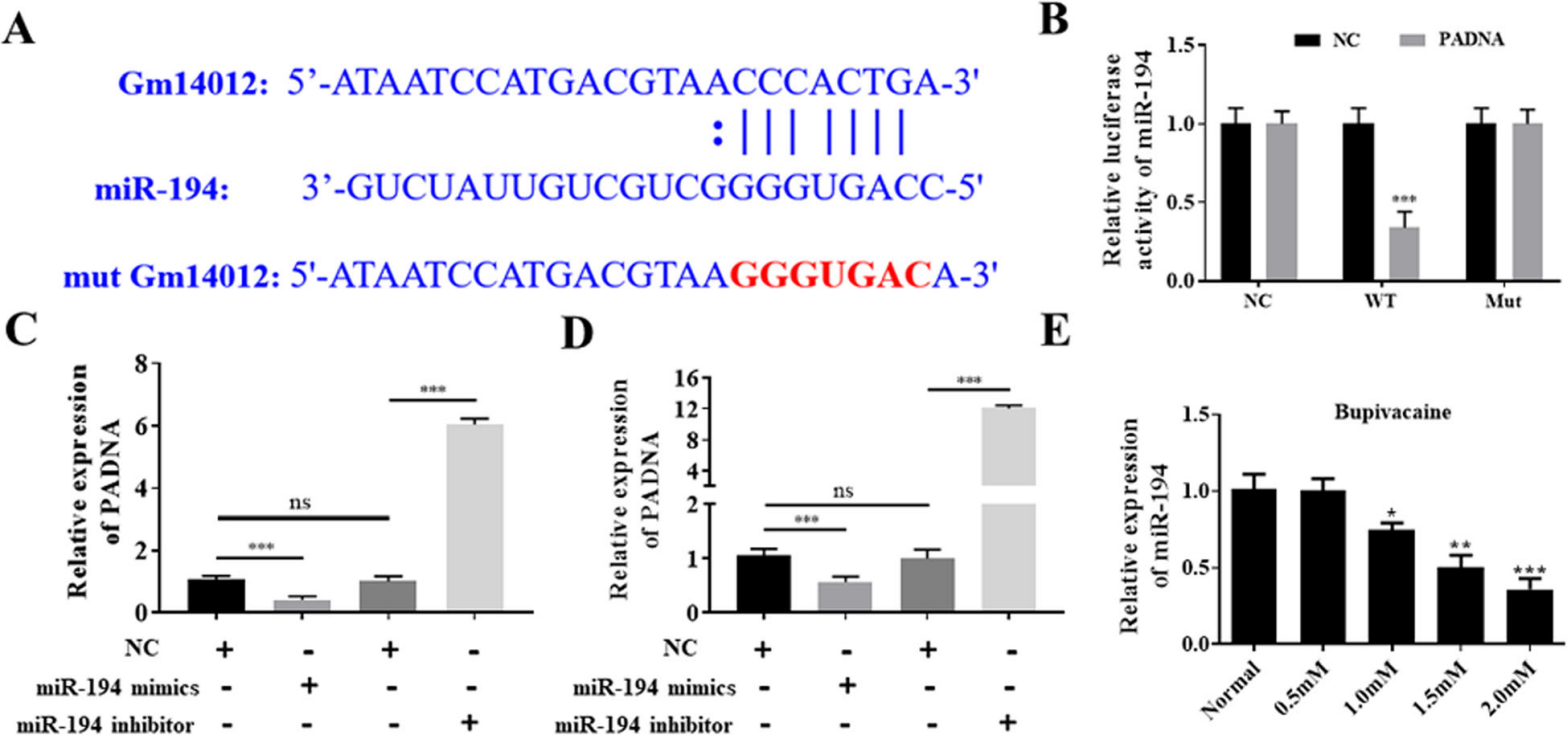
miR-194 is the direct target of lincRNA PADNA. **a** The predicted binding sequence of lincRNA PADNA and miR-194. **b** The relative luciferase activity was significantly reduced in DRG neurons co-transfected with wt-lincRNA PADNA vector and miR-194 mimics than in DRG neurons co-transfected with mut-lincRNA PADNA vector or empty vector or mimic NC. **c** Overexpression of miR-194 distinctly reduced the expression of lincRNA PADNA in DRG neurons. **d** Overexpression of miR-194 distinctly reduced the expression of lincRNA PADNA in HEK293 cells. **e** The relative expression of miR-194 was manifestly reduced with the increase of concentration of bupivacaine. Data are Mean ± SD with *n* = 3 independent biological cultures. *p < 0.05 and ** *p* < 0.01

Long noncoding RNAs often act as sponge RNAs to bind with miRNAs to mediate different functions in numerous processes. To investigate the possible mechanism of lincRNA PADNA, we first used miRDNA to predict the potential binding targets of lincRNA PADNA. As shown in Fig. 3a, miR-194 was predicted to bind with lincRNA PADNA. Next, we used dual-luciferase reporter assays to verify whether lincRNA PADNA can bind with miR-194. Our results suggested that the relative luciferase activity of miR-194 was significantly reduced in the wt-lincRNA PADNA group, while no significant difference was detected in the mut-lincRNA PADNA group, the relative decrease was about 1.0 ± 0.1, 0.34 ± 0.05 (Fig. 3b). Thus, the above results preliminarily identified miR-194 as a target of lincRNA PADNA. Next, we detected the expression of lincRNA PADNA under different conditions. Overexpression of miR-194 markedly reduced the expression of lincRNA PADNA and knockdown of miR-194 increased the expression of PADNA, the relative values are 1.05 ± 0.08, 0.41 ± 0.04, 1.00 ± 0.09, 6.04 ± 0.13 (Fig. 3c). Similar results were also obtained in HEK293 cells, the relative values are 1.02 ± 0.1, 0.56 ± 0.04, 1.03 ± 0.08, 12.04 ± 0.21 (Fig. 3d). Moreover, we also analyzed the expression of miR-194 in bupivacaine-treated cells. Our results revealed that the expression of miR-194 was clearly reduced with increasing concentrations of bupivacaine and reached its lowest level at 2.0 mM bupivacaine, the relative decrease was about 1.01 ± 0.07, 1.00 ± 0.07, 0.75 ± 0.06, 0.51 ± 0.05, 0.36 ± 0.06 (Fig. 3e). The above results revealed that lincRNA PADNA could negatively regulate the expression of miR-194.

### FBXW7 is the direct target of miR-194

**Fig. 4:**
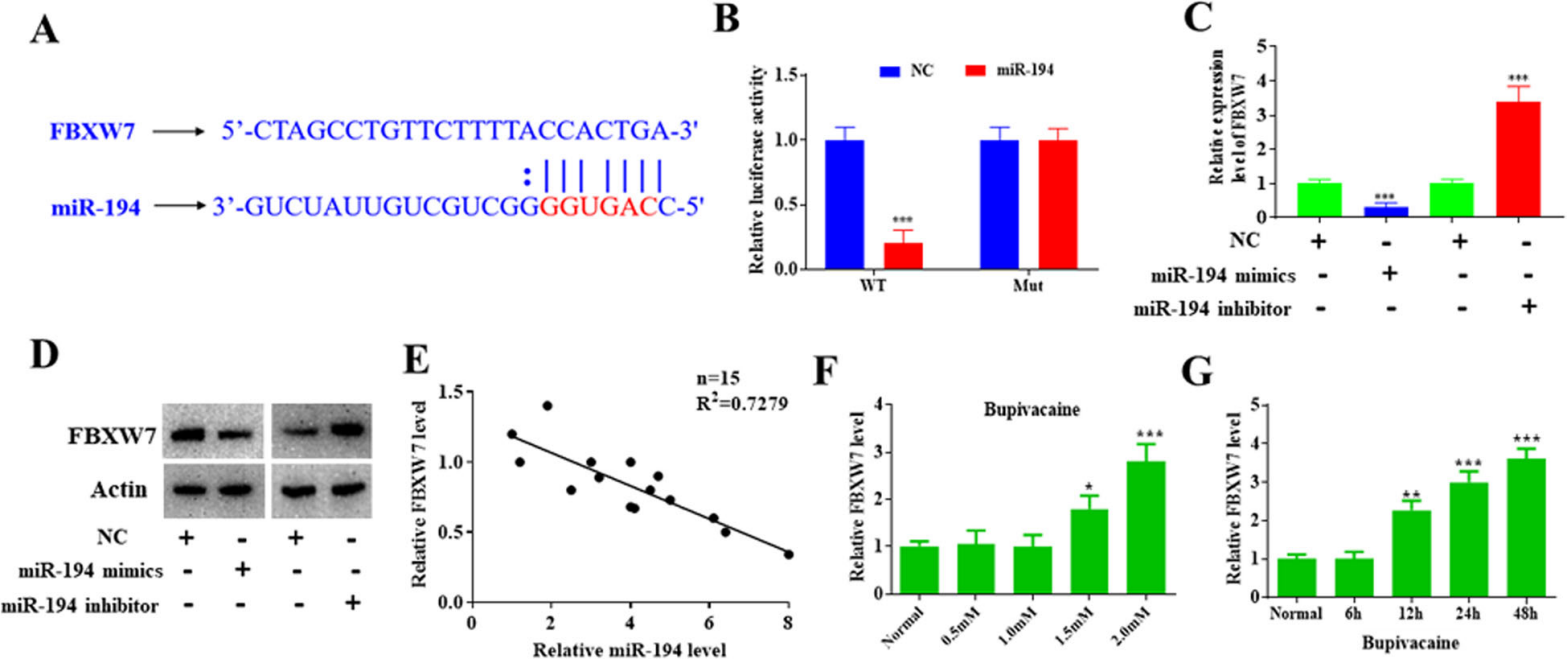
FBXW7 is the direct target of miR-194. **a** The predicted binding sequence of the 3’UTR of FBXW7’s mRNA and miR-194. **b** The relative luciferase activity was significantly reduced in DRG neurons co-transfected with wt-lincRNA PADNA vector and miR-194 mimics than in DRG neurons co-transfected with mut-lincRNA PADNA vector or empty vector or mimic NC. **c** miR-194 negatively regulate the expression of FBXW7. **d** The expression of FBXW7 protein was significantly decreased in DRG neurons treated with miR-194 than in DRG neurons treated with mimic NC; while the expression of FBXW7 protein was significantly increased in DRG neurons treated miR-194 inhibitors than in DRG neurons treated with inhibitor NC. **e** RT-PCR revealed the expression of FBXW7 mRNA is negatively correlated with the expression of miR-194. **f** The expression of FBXW7 mRNA was significantly upregulated with the elevation of concentration of bupivacaine. **g** The expression of FBXW7 mRNA was significantly upregulated with the extension of bupivacaine treatment. Cells were treated with 1 mM bupivacaine and expression of FBXW7 was measured under different time points. Data are Mean ± SD with *n* = 3 independent biological cultures. *p < 0.05 and ** *p* < 0.01

To further study the mechanism of miR-194 involved in bupivacaine-induced neurotoxicity, we used StarBase2.0 to predict the target of miR-194. We found that FBXW7 was predicted as the direct target of miR-194. Previous studies have also shown that FBXW7 is regulated by miR-194. However, the role of miR-194 and FBXW7 in bupivacaine-induced neurotoxicity remains unknown. Thus, we performed a comprehensive analysis to analyze their relationship. The binding sequence is shown in Fig. 4a. A dual-luciferase reporter assay revealed that the relative luciferase activity of wt-FBXW7 was significantly reduced in the miR-194 group, the relative values are 1.0 ± 0.09, 0.2 ± 0.04 (Fig. 4b). Overexpression of miR-194 markedly reduced the expression of FBXW7 and while silencing miR-194 reduced its expression, the relative values are 1.00 ± 0.05, 0.32 ± 0.03, 1.00 ± 0.1, 3.41 ± 0.35 (Fig. 4c). We also performed a western blot assay to analyze the protein level of FBXW7. Our results suggested that the protein level of FBXW7 was decreased in the miR-194 overexpression group (Fig. 4d). Figure 4e shows the negative correlation between miR-194 and FBXW7. In addition, we also analyzed the expression of FBXW7 in bupivacaine-treated cells. The expression of FBXW7 was increased with the increase in concentration and time course of bupivacaine and reached the highest level at 2.0 mM bupivacaine, the relative values are 1.01 ± 0.12, 1.06 ± 0.11, 1.00 ± 0.1, 1.82 ± 0.13, 2.83 ± 0.17 (Fig. 4f, g). Thus, our study provides evidence that miR-194 negatively regulates the expression of FBXW7.

### The lincRNA PADNA/miR-194/FBXW7 axis in bupivacaine-induced neurotoxicity

**Fig. 5:**
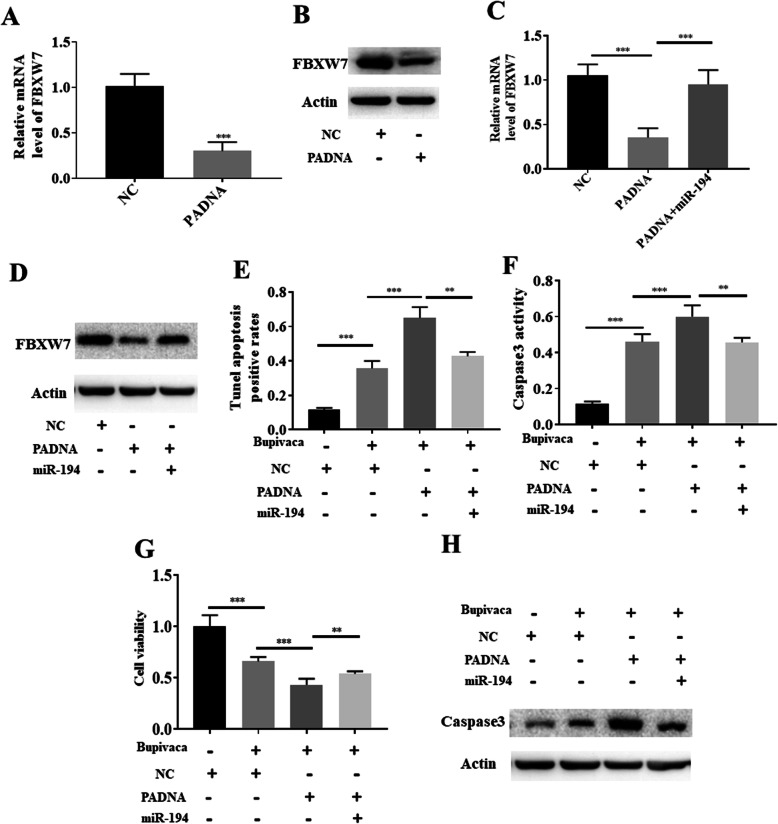
LincRNA PADNA/miR-194/FBXW7 axis in bupivacaine-induced neurotoxicity. **a** Knockdown of lincRNA PADNA significantly reduced the expression of FBXW7 mRNA in DRG neurons. **b** Knockdown of lincRNA PADNA significantly reduced the expression of FBXW7 protein in DRG neurons. **c**, **d** Knockdown of miR-194 reversed the expression of FBXW7 in DRG neurons which is inhibited by knockdown of lincRNA PADNA. **e** Knockdown of miR-194 reversed the up-regulated percentage of apoptotic of DRG neurons which is induced by the knockdown of lincRNA PADNA. Cells were treated with 1 mM bupivacaine. **f** Downregulated of miR-194 reversed the up-regulated caspase3 activity of DRG neurons which is induced by the knockdown of lincRNA PADNA. Cells were treated with 1 mM bupivacaine. **g** Downregulated of miR-194 reversed the down-regulated cell viability of DRG neurons which is induced by the knockdown of lincRNA PADNA. Cells were treated with 1 mM bupivacaine. **h** Downregulated of miR-194 reversed the up-regulated expression of caspase3 protein in DRG neurons which is induced by the knockdown of lincRNA PADNA. Cells were treated with 1 mM bupivacaine. Data are Mean ± SD with *n* = 3 independent biological cultures. *p < 0.05 and ** *p* < 0.01

We performed comprehensive analysis to further verify the lincRNA PADNA/miR-194/FBXW7 pathway. The expression of FBXW7 was downregulated in the lincRNA PADNA knockdown group, as demonstrated by qPCR, the relative values are 1.01 ± 0.1, 0.31 ± 0.07 (Fig. 5a). Similar results were obtained by western blotting suggesting that lincRNA PADNA positively regulates FBXW7 (Fig. 5b). Considering the above results, we knocked down miR-194 and PADNA and then detected the expression of FBXW7. Interestingly, the expression of FBXW7 was increased in the cotransfection group as demonstrated qPCR and western blot assays, the relative values are 1.00 ± 0.1, 0.35 ± 0.06, 0.95 ± 0.07 (Fig. 5c, d). Thus, knockdown of miR-194 could block the effect of lincRNA PADNA. Therefore, a rescue experiment was performed to verify the relationship between miR-194 and FBXW7. TUNEL assays revealed that cotransfection of miR-194 and PADNA could reduce the apoptotic effect compared with knockdown of lincRNA PADNA, the relative values are 0.11 ± 0.01, 0.35 ± 0.04, 0.6 ± 0.06, 0.41 ± 0.04 (Fig. 5e). In addition, caspase3 activity and cleaved caspase3 levels were decreased, the relative values are 0.11 ± 0.02, 0.46 ± 0.04, 0.61 ± 0.06, 0.45 ± 0.02 (Fig. 5f, h). Moreover, cell viability was increased in the cotransfection group compared with the lincRNA PADNA knockdown group, the relative values are 1 ± 0.11, 0.66 ± 0.04, 0.43 ± 0.06, 0.59 ± 0.09 (Fig. 5g). Through rescue experiments, we proved that the lincRNA PADNA/miR-194/FBXW7 axis plays an important role in bupivacaine-induced neurotoxicity.

## Discussion

The current research demonstrated that the expression of lincRNA PADNA was significantly increased in a time- and dose-dependent manner. LincRNA PADNA could prevent bupivacaine-induced neurotoxicity through the miR-194/FBXW7 axis, which may be used as a new target for inhibiting or reversing bupivacaine-induced neurotoxicity.

Anesthetic-induced neurotoxicity is one of the adverse drug reactions (ADRs) of bupivacaine that can cause permanent and irreversible neurological complications. LncRNAs have been found to play a role in anesthetic-induced neurotoxicity. For example, propofol was reported to induce neuroapoptosis in the hippocampus, with differential expression of 159 lncRNAs and 100 mRNAs (fold change ±2.0, *P* < 0.05) (Logan et al. 2018). In the current research, we established a bupivacaine-induced neurotoxicity model by treating DRG neurons with various concentrations of bupivacaine (0.5, 1.0, 1.5 or 2.0 mM) and different durations (6 h, 12 h, 24 h, or 48 h) and we found that the expression of lincRNA PADNA was significantly increased in a time- and dose-dependent manner. To further investigate the role of lincRNA PADNA, we constructed a knockdown vector to assess the function of lincRNA PADNA and found that knockdown of lincRNA PADNA significantly promoted cell death and reduced cell viability after exposure to bupivacaine. Our experiments demonstrated that lincRNA PADNA played a protective role in bupivacaine-induced neurotoxicity.

LncRNAs are extensively reported to be involved in the regulation of gene transcription (Bajolle et al. 2009), epigenetic regulation and especially posttranscriptional regulation (Ponting et al. 2009). Previous studies have reported that lncRNAs can function as competing endogenous RNAs to sponge miRNAs in various diseases (Liu et al. 2014; Luan et al. 2018). In the current research, we used bioinformatics analysis to predict the possible targets of lincRNA PADNA and found that miR-194, which has been reported to play a role in inhibiting malignant tumor progression, may be a direct target of lincRNA PADNA. Although research has indicated that miR-194 could inhibit intervertebral disc degeneration, the role of miR-194 in anesthetic-induced neurotoxicity remains unclear. To further explore the possible molecular biological function of miR-194, we first conducted a dual-luciferase reporter assay and found that the relative luciferase activity was significantly reduced in HEK cells cotransfected with the wt-lincRNA PADNA vector and miR-194 mimic compared with the mut-lincRNA PADNA group. Thus, the above results preliminarily identified miR-194 as the target of lincRNA PADNA. Next, we found that overexpression of miR-194 markedly reduced the expression of lincRNA PADNA and vice versa. Moreover, we also analyzed the expression of miR-194 in bupivacaine-treated cells, and our results revealed that the expression of miR-194 was clearly reduced with increasing concentrations of bupivacaine and reached its lowest level at 2.0 mM bupivacaine. The above results revealed that lincRNA PADNA could negatively regulate the expression of miR-194 in the setting of bupivacaine-induced neurotoxicity.

To further verify the role of the lincRNA PADNA/miR-194/FBXW7 pathway in bupivacaine-induced neurotoxicity, we conducted a comprehensive analysis and found that the expression of FBXW7 was downregulated in the lincRNA PADNA knockdown group. Cotransfection of miR-194 and lincRNA PADNA reversed the expression of FBXW7. Rescue experiments revealed that cotransfection of miR-194 and lincRNA PADNA reduced the effect induced by PADNA.

## Conclusion

Therefore, the results of comprehensive studies are in accordance with our hypothesis, and it is reasonable to believe that lincRNA PADNA could prevent bupivacaine-induced neurotoxicity by sponging miR-194 and inhibiting its function of targeting FBXW7.

## Data Availability

The dataset used and/or analyzed during the current study are available from the corresponding author on reasonable request.

## Abbreviations

CNS: Central nervous system
ADRs: Adverse drug reactions
ncRNAs: Noncoding RNAs
PADNA: Protect cell death RNA
FBXW7: F-box and WD repeat domain containing 7
WT: Wide type
UTR: Untranslated region
Mut: Mutant
MTT: 3-(4,5-dimethylthiazol-2-yl)-2,5-diphenyltetrazolium bromide
TUNEL: Terminal deoxynucleotidyl transferase-mediated dUTP nick end-labeling
